# MicroRNA Expression Profiling of Bone Marrow–Derived Proangiogenic Cells (PACs) in a Mouse Model of Hindlimb Ischemia: Modulation by Classical Cardiovascular Risk Factors

**DOI:** 10.3389/fgene.2020.00947

**Published:** 2020-08-21

**Authors:** Michel Desjarlais, Sylvie Dussault, José Carlos Rivera, Sylvain Chemtob, Alain Rivard

**Affiliations:** ^1^Department of Medicine, Centre Hospitalier de l’Université de Montréal (CHUM) Research Center, Montréal, QC, Canada; ^2^Department of Ophthalmology, Maisonneuve-Rosemont Hospital Research Center, University of Montréal, Montréal, QC, Canada; ^3^Departments of Pediatrics, Ophthalmology and Pharmacology, Centre Hospitalier Universitaire Sainte-Justine Research Center, Montréal, QC, Canada

**Keywords:** microRNA (miR), proangiogenic cell (PAC), cardiovascular risk factors, hindlimb ischemia, neovascularization

## Abstract

**Background:**

Classical cardiovascular risk factors (CRFs) are associated with impaired angiogenic activities of bone marrow–derived proangiogenic cells (PACs) related to peripheral artery diseases (PADs) and ischemia-induced neovascularization. MicroRNAs (miRs) are key regulators of gene expression, and they are involved in the modulation of PAC function and PAC paracrine activity. However, the effects of CRFs on the modulation of miR expression in PACs are unknown.

**Aims and Methods:**

We used a model of hindlimb ischemia and next-generation sequencing to perform a complete profiling of miRs in PACs isolated from the bone marrow of mice subjected to three models of CRFs: aging, smoking (SMK) and hypercholesterolemia (HC).

**Results:**

Approximately 570 miRs were detected in PACs in the different CRF models. When excluding miRs with a very low expression level (<100 RPM), 40 to 61 miRs were found to be significantly modulated by aging, SMK, or HC. In each CRF condition, we identified downregulated proangiogenic miRs and upregulated antiangiogenic miRs that could contribute to explain PAC dysfunction. Interestingly, several miRs were similarly downregulated (e.g., miR-542-3p, miR-29) or upregulated (e.g., miR-501, miR-92a) in all CRF conditions. *In silico* approaches including Kyoto Encyclopedia of Genes and Genomes and cluster dendogram analyses identified predictive effects of these miRs on pathways having key roles in the modulation of angiogenesis and PAC function, including vascular endothelial growth factor signaling, extracellular matrix remodeling, PI3K/AKT/MAPK signaling, transforming growth factor beta (TGFb) pathway, p53, and cell cycle progression.

**Conclusion:**

This study describes for the first time the effects of CRFs on the modulation of miR profile in PACs related to PAD and ischemia-induced neovascularization. We found that several angiogenesis-modulating miRs are similarly altered in different CRF conditions. Our findings constitute a solid framework for the identification of miRs that could be targeted in PACs in order to improve their angiogenic function and for the future development of novel therapies to improve neovascularization and reduce tissue damage in patients with severe PAD.

## Introduction

Peripheral artery disease (PAD) is a major health problem. It affects more than 200 million men and women worldwide and is the third leading cause of cardiovascular morbidity after coronary artery disease and stroke (Criqui and Aboyans, 2015). PAD is associated with intermittent claudication and impaired mobility. In its more severe form, PAD leads to chronic ischemia with rest pain, non-healing ulcers, tissue necrosis, gangrene, and lower limb amputation. Improving the natural capacity of the organism to develop new blood vessels (neovascularization) is an attractive strategy to promote perfusion of affected muscles, reduce pain, and avoid ischemic tissue damage and limb amputation (Losordo and Dimmeler, 2004; Cooke and Losordo, 2015). Neovascularization involves the development of collateral vessels (arteriogenesis), as well as the activation, proliferation, and migration of mature endothelial cells that will extend the preexisting vascular network (angiogenesis) (D’Amore and Thompson, 1987). However, it has now been demonstrated that postnatal neovascularization also depends on the action of bone marrow–derived proangiogenic cells (PACs) (Asahara et al., 1997; Asahara et al., 1999). PACs, initially described as early outgrowth endothelial progenitor cells (EPCs), were shown to reach sites of ischemia where they can promote neovascularization mainly through paracrine secretion of angiogenic factors and cytokines (Urbich and Dimmeler, 2004).

The administration of autologous mononuclear cells, including PACs, represents a possible therapeutic option for PAD patients with critical limb ischemia (CLI) (Cooke and Losordo, 2015). However, although positive results have been reported in preclinical studies and small clinical trials, several placebo-controlled randomized studies showed no advantage from bone marrow mononuclear cell administration over placebo (Peeters Weem et al., 2015). One possible explanation for these disappointing results is that the administered autologous cells might be dysfunctional in PAD patients who present several comorbid conditions. In fact, it has been demonstrated that classical cardiovascular risk factors (CRFs) involved in the development of atherosclerosis and PAD are also associated with impaired number and/or functional activities of PACs in humans (Vasa et al., 2001; Tepper et al., 2002; Hill et al., 2003; Chen et al., 2004; Michaud et al., 2006). However, the specific mechanisms leading to PAC dysfunction in these pathological conditions are poorly understood.

MicroRNAs (miRNAs or miRs) represent a novel class of endogenous non-coding small RNA molecules (20–25 nucleotides) that regulate a wide range of physiological and pathological processes, including angiogenesis (Urbich et al., 2008; Suarez and Sessa, 2009; Landskroner-Eiger et al., 2013). miRs can impact on stem/progenitor cell differentiation and function during cardiovascular repair responses (Jakob and Landmesser, 2012). In addition, miRs can be released by PACs to protect against ischemia and stimulate neovascularization in different tissues (Cantaluppi et al., 2012; Ranghino et al., 2012). Therefore, dysregulation of miR expression could contribute to explain the impaired functional activities of PACs in PAD patients with CRFs. Here, in a mouse model of PAD, we used next-generation sequencing (NGS) to study PAC miR expression profile in the context of three key CRFs: smoking (SMK), hypercholesterolemia (HC), and aging. Our results indicate that CRFs lead to important modulations in the expression of miRs in PACs, including several miRs predicted to be involved in the response to ischemia, angiogenesis, and postnatal neovascularization. Moreover, overlaps in the modulation of miRs by different CRFs might define a set of miRs with angiogenic/antiangiogenic properties that could be targeted in order to improve the function of PACs and promote therapeutic neovascularization in PAD patients with CLI.

## Materials and Methods

### Murine Hindlimb Ischemia and Classical CRF Models

The protocol was approved by the Comité Institutionnel de Protection des Animaux of the Center Hospitalier de l’Université de Montréal. Unilateral hindlimb ischemia was surgically induced by removing the femoral artery under anesthesia with 2% isoflurane (Desjarlais et al., 2017; Desjarlais et al., 2019) and PACs were isolated from the bone marrow 2 days after ischemia. Four groups of mice were studied. The effect of aging was studied in 16-month-old C57BL/6 mice (aging). Young 2- to 3-month-old C57BL/6 mice were used as controls (CTL). The effect of SMK was studied in 2- to 3-month-old C57BL/6 mice exposed or not (CTL) to cigarette smoke (two cigarettes, twice a day) via an SMK machine starting 14 days prior to surgery (Dhahri et al., 2017). Commercial cigarettes (Player’s Plain, tar: 17 mg, nicotine: 1.5 mg, carbon monoxide: 12 mg) were used. The effect of HC was studied in 2- to 3-month-old hypercholesterolemic ApoE^–/–^ mice (C57BL/6 background) purchased from Jackson Laboratory (Bar Harbor, ME, United States) and put on a Western-type diet (1.25% cholesterol, 15% cocoa butter, 0.5% sodium cholate; Teklad 90221) beginning 5 weeks before the surgery (Desjarlais et al., 2017). Two- to 3-month-old normocholesterolemic C57BL/6 mice were used as CTL.

### PAC Isolation and Characterization

Two days after hindlimb ischemia, bone marrow mononuclear cells were isolated from the femora and tibiae by flushing the bone marrow cavities using culture medium and kept on fibronectin-coated (Sigma, St. Louis, MO, United States) plates. After 4 days in culture, non-adherent cells were removed by thorough washing with phosphate-buffered saline (PBS). Adherent cells were stained with DAPI (0.5 mg/mL; Life Technologies); 1,10-dictadecyl-3,3,30,30 acetylated low-density lipoprotein (DiI-acLDL, 2.5 mg/mL for 1 h; Life Technologies); and fluorescein isothiocyanate (FITC)–labeled lectin BS-1 (*Bandeiraea simplicifolia*, 10 mg/mL for 1 h; Sigma). Bone marrow PACs were characterized as adherent cells that were positive for both DiI-acLDL uptake and lectin binding as previously described (Desjarlais et al., 2017; Dhahri et al., 2017; Desjarlais et al., 2019).

### PAC Migration Assay

Proangiogenic cells migration was assessed using a modified Boyden chamber assay. Twenty thousand cells in growth factor–deprived medium were added to the upper chamber of a Transwell insert (pore size 8 μm; Corning, Corning, NY, United States) coated with 0.1% gelatin. The inserts were placed in a 24-well plate containing medium 200 with 50 ng/mL vascular endothelial growth factor (VEGF). After incubation for 6 h at 37°C, the cells that did not migrate were removed by wiping the upper surface with an absorbent tip. The migrant cells were fixed for 10 min with 3.7% formaldehyde and stained with hematoxylin. The number of cells that migrated was counted in three different representative high-power (200×) fields per insert. All experiments were performed in duplicate.

### PAC Adhesion to an Endothelial Monolayer

A monolayer of human umbilical vein endothelial cells (HUVECs) (passages 4–6) was prepared in 24-well plates. HUVECs were pretreated for 16 h with tumor necrosis factor α (1 ng/mL; BD Biosciences), fixed and stained with DAPI (0.5 mg/mL; Life Technologies). PACs were labeled with DiI-AcLDL, and 15,000 cells were added to each well (2 wells/mouse) and incubated for 3 h at 37°C. Non-attached cells were gently removed with PBS, and adherent PACs were fixed with 2% paraformaldehyde and counted in three random fields per well.

### miR Isolation and NGS Analyses

PACs were isolated from the different groups of mice 2 days after surgery, and total RNA was extracted from PACs after 4 days in culture using the Ambion mirVana^TM^ miR isolation kit (Life Technologies) according to the manufacturer’s protocol. RNA quality was validated with the BioAnalyzer Nano (Agilent) using an RNA pico chip, and all samples had an RIN greater than eight. The amount of RNA we could recover from PACs isolated from one mouse was limited. Therefore, in order to optimize the quantity of RNA and also to avoid excessive cost, we pooled the RNA samples in each group before performing NGS. This strategy was recently shown to be a good option to optimize the cost and maintain the power for differential gene expression analysis (Assefa et al., 2020). Equal amounts of RNA samples (4–6/group) were pooled in each experimental condition: CTL, aging, SMK, and HC. Final RNA concentrations were then determined in each group, and an equal quantity of total RNA was used for library preparation. Library preparation was done with the QIAseq miR Library Kit (Qiagen). Sequencing was performed on a NextSeq 500 (Illumina), obtaining approximately 5 million reads per sample. The QIAseq miRNA sequencing files were uploaded to the GeneGlobe^®^ Data Analysis center. To annotate the insert sequences, a unique sequence set was made for all readsets/samples. Following this, a sequential alignment strategy was followed to map to different databases (perfect match to miRBase mature, miRBase hairpin, non-coding RNA, mRNA and otherRNA, and ultimately a second mapping to miRBase mature, where up to two mismatches are tolerated) using bowtie (bowtie-bio.sourceforge.net/index.shtml). At each step, only unmapped sequences pass to the next step. Read counts for each RNA category (miRBase mature, miRBase hairpin, piRNA, tRNA, rRNA, mRNA, and otherRNA) were calculated from the mapping results (miRNA_Reads, hairpin_Reads, piRNA_Reads, etc.). miRBase V21 was used for miRNA. A mouse-specific miRBase mature database was used, and all remaining unmapped sequences were aligned to the mouse genome (Genome Reference Consortium GRCm38). Raw read counts obtained in each library are presented in Supplementary Table 1. Normalization by library size was then performed, and values expressed as reads per million reads mapped (RPM). We focused on miRs, with an expression level of at least 100 RPM, and differential gene expression levels were expressed as fold change between the CRF condition and the CTL (CRF/CTL). miRs were considered to be significantly upregulated or downregulated if the fold change value was greater than 1.6 for upregulated miRs and lower than 0.8 for downregulated miRs. Next-generation sequencing (NGS) data have been deposited and are available on NCBI Gene Expression Omnibus, GSE151609.

### Identification of Predictive Targets/Pathways of miRs Modulated by CRFs in PACs

Predicted and validated target genes involved in angiogenesis, inflammation, oxidative stress, cell survival, apoptosis, senescence, and other pathways potentially linked to PAC dysfunction were identified in the different CRF conditions using the bioinformatic algorithms of miRSystem database (^1^; version 20160513), which is an integrated system used to characterize the enriched functions and pathways of miRNA targets (Lu et al., 2012). In target prediction, the miRSystem database integrates two experimentally validated databases, TarBase (version 7.0) and miRecords (April 27, 2013, release), and seven target gene prediction algorithms, including DIANA-microT (version 5.0), miRanda (August 2010 release), miRBridge (April 2010 release), PicTar (March 2007 release), PITA (August 2008 release), RNA22 (version 2.0), and Targetscan (version 7.1). Kyoto Encyclopedia of Genes and Genomes (KEGG) pathways and dendrogram clusters of predictive biological Gene Ontology (GO) process-connectome of selected miRs were analyzed using DIANA tool program^2^ (Vlachos et al., 2015).

### Statistical Analysis

**FIGURE 1:**
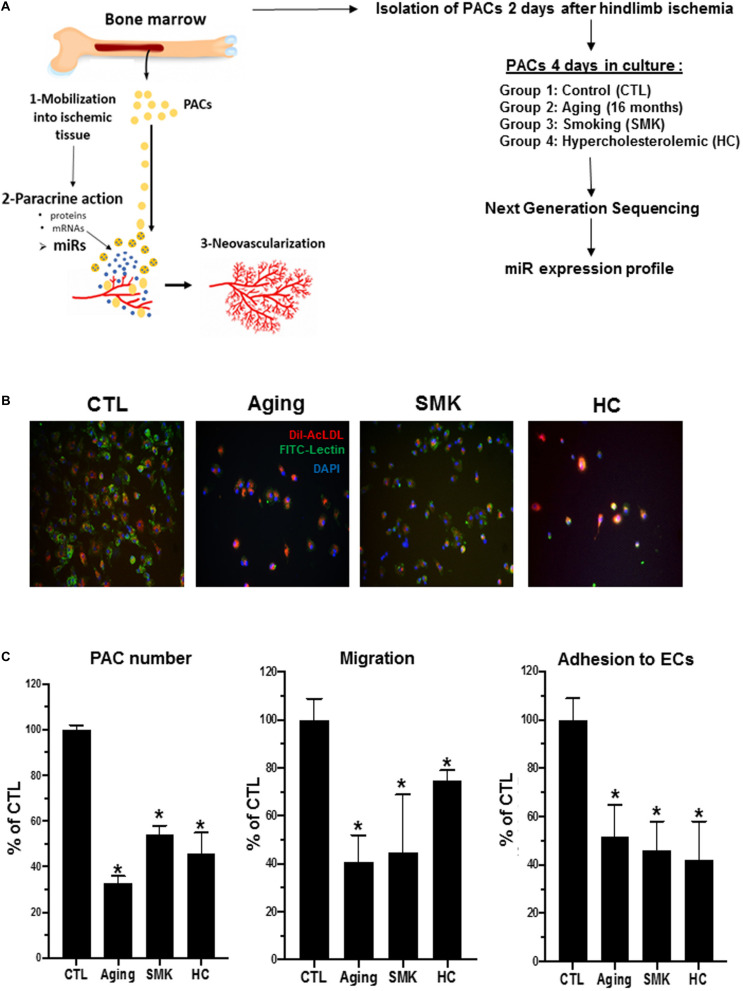
Experimental design and effect of aging, smoking (SMK), and hypercholesterolemia (HC) on the number and functional activities of PACs. **(A)** Schematic representation of PAC angiogenic function (left) and experiment design used for miR profiling in PACs (right). **(B)** Representative pictures of PACs isolated from control (CTL), aging, SMK, and HC mice at day 2 after hindlimb ischemia. PACs were cultured *ex vivo* for 4 days before staining with DAPI (blue), BS-1 lectin-FITC (green), and DiI-acLDL (red). **(C)** Quantitative analysis of PAC number, PAC migration capacity, and PAC adhesion to endothelial cells (ECs) in the different groups of mice. Data are mean ± SEM (*n* = 4–6/group). *P < 0.05 versus CTL.

Results for PAC number and function (Figure 1) are presented as mean ± SEM. Statistical significance was evaluated by a one- or two-way analysis of variance followed by a Bonferroni *post hoc* test. *P* < 0.05 was interpreted to denote statistical significance.

## Results

### Overview of miR Expression Profile in PACs Isolated From Aging, SMK, and HC Mice After Hindlimb Ischemia

**FIGURE 2:**
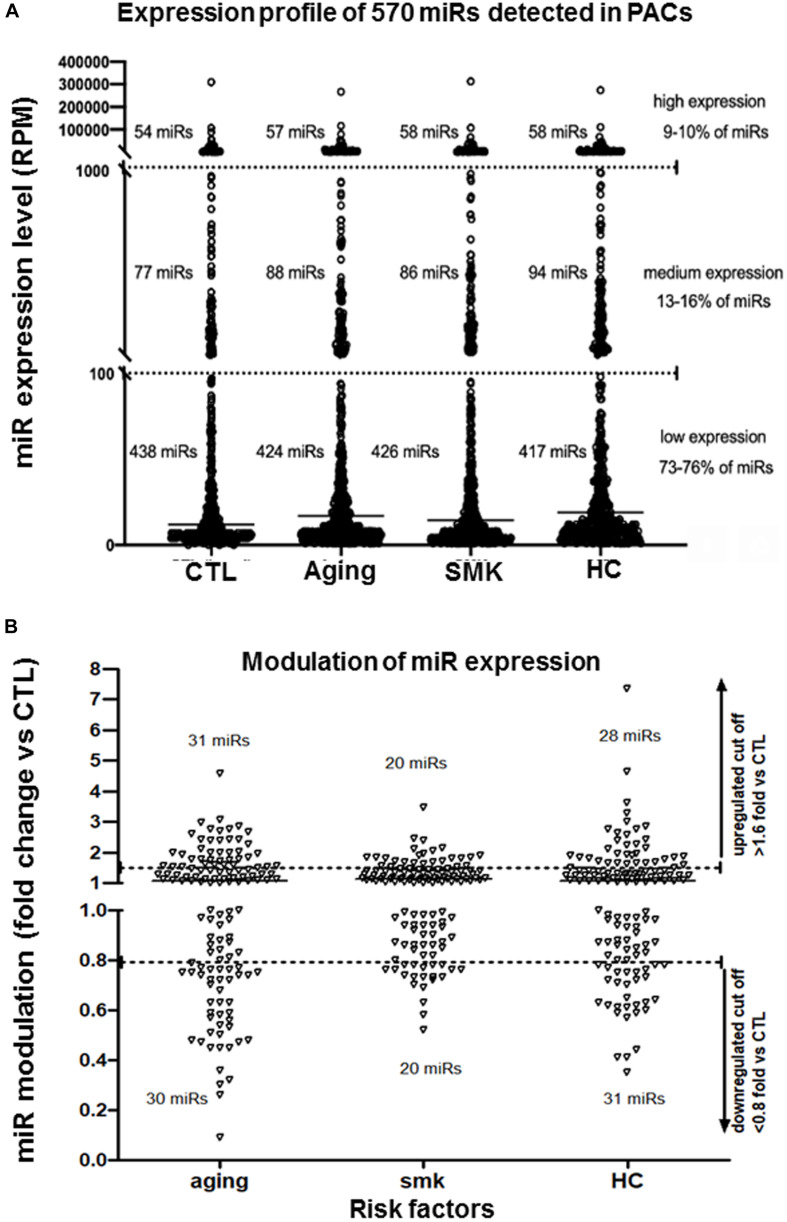
Effect of aging, smoking (SMK), and hypercholesterolemia (HC) on the global expression levels of miRs in PACs. **(A)** Global evaluation of miR expression levels [reads per million of reads mapped (RPM)] in PACs isolated 2 days after hindlimb ischemia in aging, SMK, and HC mice compared to control mice (CTL). **(B)** Specific effect of aging, SMK, and HC on the modulation of miR expression. miRs with an expression level of at least 100 RPM are shown in each condition (pool of four to six mice per condition). Arbitrary cutoff levels of 0.8-fold (for downregulated miRs) and 1.6-fold (for upregulated miRs) are indicated.

To study how modulation of miRs could affect the function of PACs in pathological conditions, we used NGS to perform a complete profile of miRs in PACs isolated from mice subjected to three models of CRFs: aging (Groleau et al., 2011), SMK (Dhahri et al., 2017), and HC (Desjarlais et al., 2017; Figure 1A). PACs were isolated from the bone marrow of the different groups of mice 2 days after hindlimb ischemia surgery and cultured *ex vivo* for 4 days. Total RNA was extracted to perform NGS miR analyses. As shown in Figures 1B,C, the number of PACs and their chemotactic migration and adhesion properties were significantly decreased compared to CTL in aging, SMK, and HC mice. Next, we quantified the number of miRs in PACs exposed to the different CRFs and detected ∼570 miRs in each group, with a similar distribution of their level of expression (abundance): 73 to 76% low expression, 13 to 16% medium expression, and 9 to 10% high expression (Figure 2A). We then focused on the most abundant and modulated miRs by establishing an arbitrary expression cutoff level of 100 reads per million of reads mapped (RPM) and a modulation cutoff level of 0.8-fold versus CTL for downregulated miRs and 1.6-fold versus CTL for upregulated miRs. We identified 40 to 61 modulated miRs in PACs in the different CRF conditions (61 miRs in aging, 40 miRs in SMK, 59 miRs in HC) with a similar number of upregulated and downregulated miRs (Figure 2B).

### Effect of Aging on the Modulation of miRs in PACs in the Context of Hindlimb Ischemia

**FIGURE 3:**
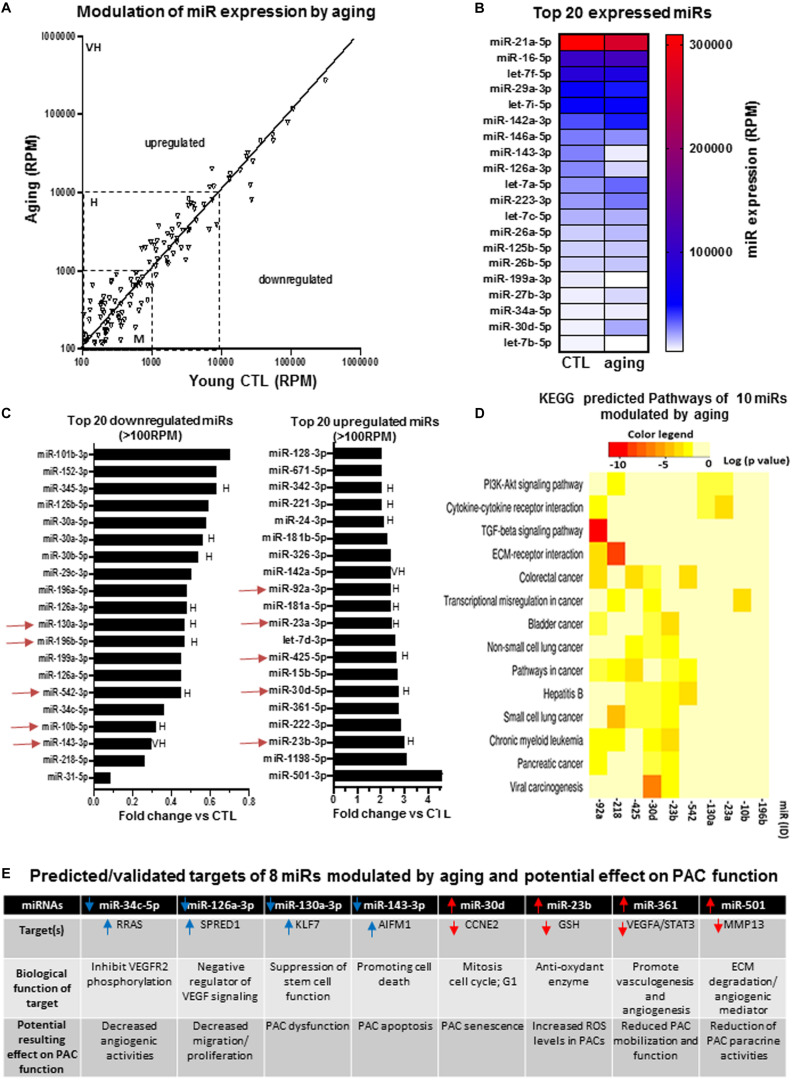
Specific effect of aging on miR expression profile in PACs and bioinformatic analyses of predictive targets/pathways potentially involved in PAC dysfunction. **(A)** Overview of miR expression distribution in PACs isolated from aging versus young (CTL) mice. **(B)** Heat map showing the effect of aging on the expression of the 20 most abundant miRs in PACs. **(C)** Overview of the top 20 downregulated and upregulated miRs in PACs in the context of aging. **(D)** KEGG analysis to identify predicted pathways involved in 10 highly expressed/highly modulated miRs [red arrows in (C)]. **(E)** Predicted/validated targets of eight miRs modulated by aging and potentially associated with PAC dysfunction. M, H, and VH indicate miR with medium (100–1,000 RPM), high (1,000–10,000 RPM), and very high (>10,000 RPM) expression, respectively.

We specifically looked at the expression profile of miRs in PACs isolated from the bone marrow of aging (16 months old) versus young (2 months old) mice at day 2 after hindlimb ischemia. Figure 3A confirms that several miRs are modulated by aging in PACs. The modulation is often seen in miRs showing moderate (100–1,000 RPM) or high (1,000–10,000 RPM) expression levels compared to those with very high expression levels (>10,000 RPM). For example, the five miRs exhibiting levels of expression greater than 100,000 RPM (miR-21a-5p, miR-16-5p, let-7f, miR-29a-3p and let-7i-5p) are not modulated by aging (Figures 3A,B). We next focused on miRs that showed the highest level of modulation (upregulation or downregulation) by aging (Figure 3C). Among downregulated miRs, four miRs (miR-31-p, miR-218-5p, miR-143-5p, and miR-10b-5p) showed greater than 70% suppression. On the other hand, three miRs were upregulated by 300% or more (miR-501-3p, miR-1198-5p, and miR-23b-3p). To better understand the potential biological effects of miR modulation by aging, we used KEGG analysis to identify predicted pathways involved in 10 highly expressed/highly modulated miRs (red arrows in Figure 3C). As seen in Figure 3D, the bioinformatic algorithm suggests that 14 pathways/processes are affected by these miRs. Interestingly, most of these pathways are involved in carcinogenesis, a condition that is linked to angiogenesis. Other key angiogenic pathways such as PI3K/AKT and transforming growth factor beta (TGFb) signaling are also predicted to be modulated by these miRs (Figure 3D). In order to begin understanding the role that individual miR might have on PAC function in the context of aging, we identified eight modulated miRs with predicted targets involved in angiogenesis or related processes such as inflammation, senescence, apoptosis, and oxidative stress. As seen in Figure 3E, downregulation of miR-34c and miR-126a-3p, two proangiogenic miRs, could negatively regulate VEGF signaling through upregulation of RRAS (Sawada et al., 2015) and SPRED1 (Fish et al., 2008), respectively, resulting in decreased angiogenic properties of PACs. Downregulation of miR-130a-3p could lead to stem cell dysfunction via increased KLF7 expression (Schuettpelz et al., 2012), whereas downregulation of miR-143-3p could decrease PAC survival by increasing the level of the proapoptotic factor AIFM1 (Bano and Prehn, 2018). On the other hand, upregulation of miR-30d and miR-23b could promote cell senescence and oxidative stress levels in PACs by decreasing the levels of CCNE2 (a promitotic factor) (Caldon et al., 2013) and GSH (an antioxidant enzyme) (Forman et al., 2009), respectively. The upregulation of miR-361 and miR-501 could decrease the angiogenic paracrine activity of PACs by targeting VEGF (Dal Monte et al., 2013) and MMP-13 (Kudo et al., 2012), respectively.

### Effect of Cigarette Smoke Exposure on the Modulation of miRs in PACs in the Context of Hindlimb Ischemia

**FIGURE 4:**
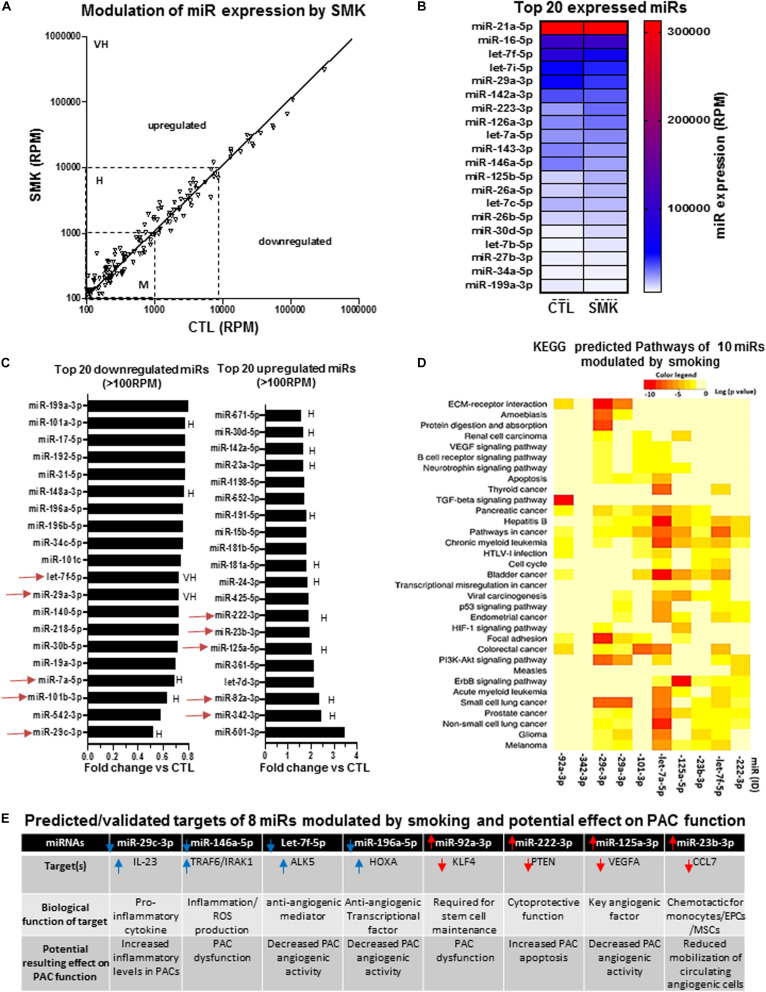
Specific effect of smoking (SMK) on miR expression profile in PACs and bioinformatic analyses of predictive targets/pathways potentially involved in PAC dysfunction. **(A)** Overview of miR expression distribution in PACs isolated from SMK versus control (CTL) mice. **(B)** Heat map showing the effect of smoking on the expression of the 20 most abundant miRs in PACs. **(C)** Overview of the top 20 downregulated and upregulated miRs in PACs in the context of smoking. **(D)** KEGG analysis to identify predicted pathways involved in 10 highly expressed/highly modulated miRs [red arrows in **(C)**]. **(E)** Predicted/validated targets of eight miRs modulated by smoking and potentially associated with PAC dysfunction. M, H, and VH indicate miR with medium (100–1,000 RPM), high (1,000–10,000 RPM), and very high (>10,000 RPM) expression, respectively.

We compared the expression profile of miRs in PACs isolated from the bone marrow of mice exposed (SMK) or not (CTL) to cigarette smoke at day 2 after hindlimb ischemia. Figure 4A confirms that several miRs are modulated by SMK in PACs. The modulation is more often seen in miRs showing moderate (100–1,000 RPM) or high (1,000–10,000 RPM) expression levels compared to those with very high expression levels (>10,000 RPM). The miRs exhibiting the highest levels of expression are not modulated by SMK (Figures 4A,B). However, in opposition to aging, few miRs were significantly reduced by SMK in PACs. Only three miRs were downregulated by >30% (miR-29c-3p, miR-542-3p, miR-7a-5p), and no miR was downregulated by more than 60% (Figure 4C). On the other hand, three miRs were increased by >225%: miR-501-3p, miR-342-3p, and miR-92a-3p. KEGG analysis was performed in 10 highly expressed/highly modulated miRs (red arrows in Figure 4C). As seen in Figure 4D, the bioinformatic algorithm suggests that 34 pathways/processes are affected by these miRs. Interestingly, key angiogenic signaling pathways such as VEGF, hypoxia-inducible factor 1 (HIF1), and TFGb are predicted to be regulated by these miRs. Individually, two miRs (miR-29c-3p and miR-146a-5p) that are downregulated by SMK have proinflammatory targets such IL-23 (Botta et al., 2018) and TRAF6/IRAK1 (Saba et al., 2014), which could increase inflammation and induce PAC dysfunction (Figure 4E). In addition, reduced expression of miR-196a-5p and let-7f-5p could, respectively, increase the levels of HOXA-5 (Liu et al., 2012; Cuevas et al., 2015) and ALK5 (Dhahri et al., 2017), two antiangiogenic factors that could negatively affect PAC function. On the other hand, four upregulated miRs could impair PAC survival and PAC mobilization. For example, miR-92a-3p targets KLF4 (which is required for stem cells maintenance) (Yu et al., 2011; Fang and Davies, 2012), miR-222 targets PTEN (a cytoprotective factor for stem cell differentiation) (Garofalo et al., 2009; Lyu et al., 2015), miR-125a-3p targets VEGF (Yang et al., 2018), and miR-23b targets the chemotactic factor CCL7 (Zhang et al., 2018; Figure 4E).

### Effect of Hypercholesterolemia on the Modulation of miRs in PACs in the Context of Hindlimb Ischemia

**FIGURE 5:**
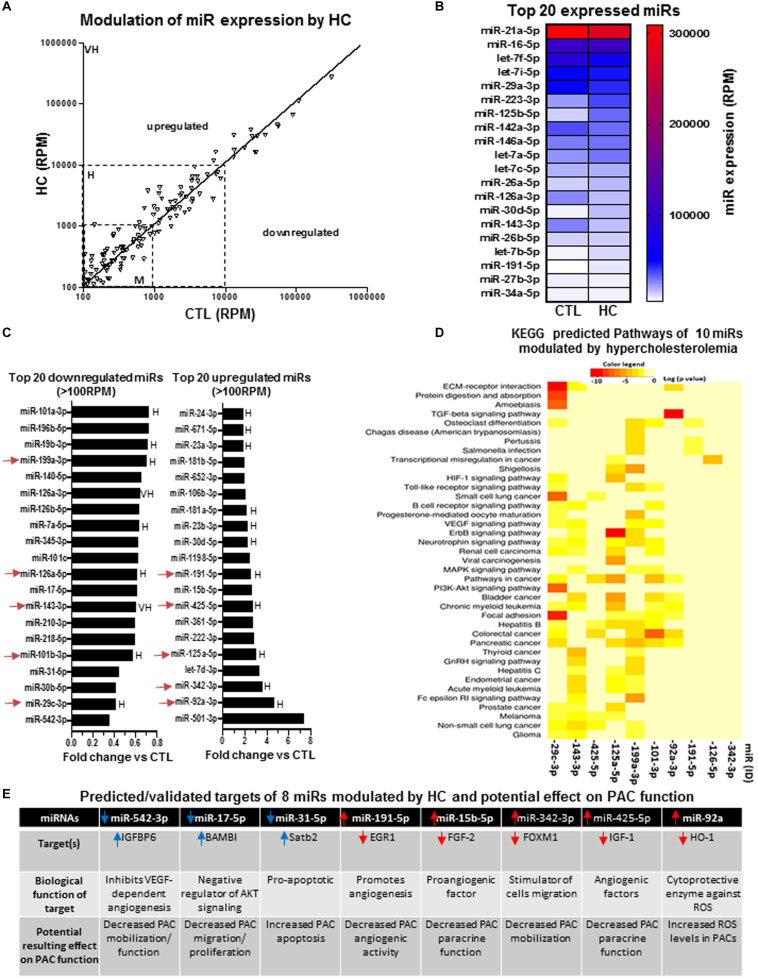
Specific effect of hypercholesterolemia on miR expression profile in PACs and bioinformatic analyses of predictive targets/pathways potentially involved in PAC dysfunction. **(A)** Overview of miR expression distribution in PACs isolated from hypercholesterolemic (HC) versus control (CTL) mice. **(B)** Heat map showing the effect of hypercholesterolemia on the expression of the 20 most abundant miRs in PACs. **(C)** Overview of the top 20 downregulated and upregulated miRs in PACs in the context of hypercholesterolemia. **(D)** KEGG analysis to identify predicted pathways involved in 10 highly expressed/highly modulated miRs [red arrows in **(C)**]. **(E)** Predicted/validated targets of eight miRs modulated by hypercholesterolemia and potentially associated with PAC dysfunction. M, H, and VH indicate miR with medium (100–1,000 RPM), high (1,000–10,000 RPM), and very high (>10,000 RPM) expression, respectively.

We next compared the expression profile of miRs in PACs isolated from the bone marrow of hypercholesterolemic (HC) or normocholesterolemic (CTL) mice at day 2 after hindlimb ischemia. Figure 5A confirms that several miRs are modulated by HC in PACs. Interestingly, contrary to aging and SMK, we identified miRs with very high expression levels that were modulated by HC. Among the top 15 miRs in abundance, two miRs (miR-223-3p and miR-125b-5p) were strongly upregulated, whereas three miRs (miR-142a-3p, miR-126a-3p, and miR-143-3p) were strongly downregulated in PACs exposed to HC conditions (Figures 5A,B). Among the top 20 miRs modulated by HC (Figure 5C), three miRs were downregulated by greater than 60% including miR-542-3p, miR-30b-5p, and the highly expressed miR-29c-3p. Four miRs were upregulated by greater than 300% including the highly expressed miR-92a-3p and miR-342-3p and the moderately expressed let-7d-3p and miR-501-3p (>700% upregulation). KEGG analysis was performed in 10 highly expressed/highly modulated miRs (red arrows in Figure 5C). As seen in Figure 5D, the bioinformatic algorithm suggests that 39 pathways/processes are affected by these miRs including VEGF, HIF1, and TFGb pathways. Individually, three miRs that are downregulated by HC and five miRs that are upregulated could contribute to PAC dysfunction (Figure 5E). For example, reduced expression of miR-542-3p could inhibit VEGF signaling by increasing IGFBP1 (a negative regulator of VRGFR2 activation) (Zhang et al., 2012; Tochigi et al., 2017). Reduced expression of miR-17-5p can increase the levels of BAMBI (Duan et al., 2019), a negative regulator of AKT signaling that is involved in PAC migration and proliferation (Zheng et al., 2007). Likewise, reduced expression of miR-31-5p could increase Satb2 levels and promote apoptotic death (Lian et al., 2018). On the other hand, upregulation of miR-15b-5p, miR-342-3p and miR-425-5p could impair PAC angiogenic properties by inhibiting the proangiogenic factors FGF-2, FOXM1, and IGF-1, respectively (Li et al., 2014; Liu et al., 2015; Schelch et al., 2018).

### Integrated Model of the Effect of CRFs on miR Modulation in PACs Related to Their Angiogenic Function

**FIGURE 6:**
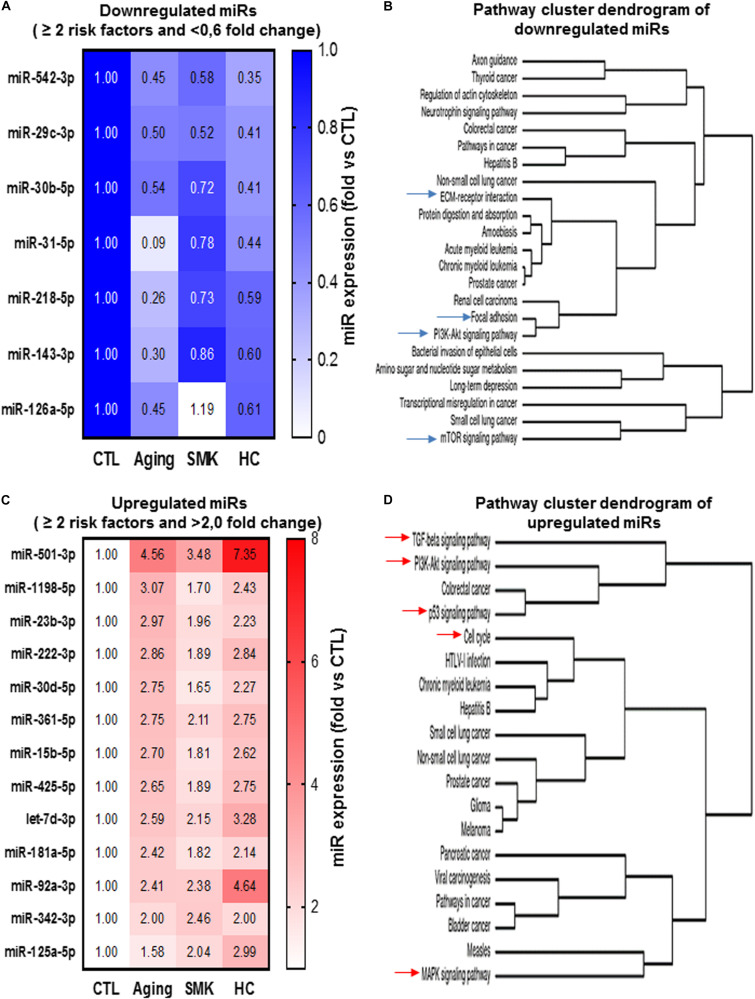
Convergent modulation effect of different CRFs on the expression of miRs in PACs. Heat maps **(A,C)** and pathway cluster dendogram analyses **(B,D)** of miRs that are downregulated (<0.6-fold versus CTL) or upregulated (>2.0-fold versus CTL) by two risk factors or more in PACs.

**FIGURE 7:**
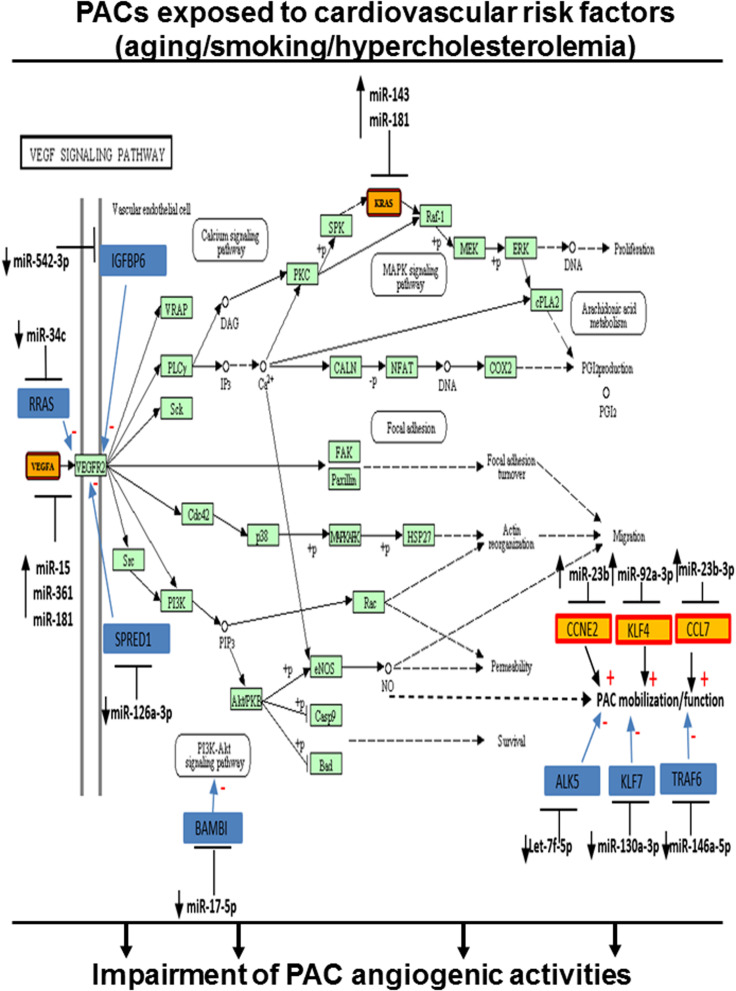
Integrated model of the effect of CRFs on miR modulation in PACs related to their angiogenic function. Connectome of predicted (yellow) and validated (blue) targets of miRs modulated by CRFs in PACs and involved in VEGF signaling and/or PAC function, based on bioinformatic algorithms (miRSystem database).

To evaluate the cumulative effect that different CRFs might have on miR modulation and PAC function, we identified miRs that were modulated similarly (upregulation or downregulation) in two or more CRF conditions. As shown in Figure 6A, seven miRs were downregulated in two or more CRF conditions, including two miRs (miR-29c-3p and miR-542-3p) that were downregulated in all three CRF conditions. We used cluster dendrogram predictive analysis (Figure 6B) to better appreciate the resulting physiological and/or pathological effects of the seven miRs that were downregulated in two or more CRF conditions. We found 23 different pathways that could be affected, including several linked to angiogenesis such as extracellular matrix (ECM)–receptor interaction, focal adhesion, PI3K/AKT, and mTOR signaling (Ramjaun and Hodivala-Dilke, 2009; Karar and Maity, 2011; Mongiat et al., 2016). On the other hand, as shown in Figure 6C, 13 miRs were upregulated in two or more CRF conditions, including two miRs (miR501-3p and miR-92a-3p) that were robustly upregulated in all three CRF conditions. Here, cluster dendrogram analysis to evaluate the global effects of the 13 miRs that were upregulated in two or more CRF conditions (Figure 6D) predicted that 19 different pathways could be affected, including several linked to angiogenesis and/or cell cycle such as TGFb, PI3K/AKT, p53, and MAPK signaling (Wu, 2004; Karar and Maity, 2011; van Meeteren et al., 2011). To gain further insight into the interrelated effects of miR modulation by CRFs in PACs, we perform a connectome analysis based on VEGF signaling, a crucial physiological pathway involved in both angiogenesis and postnatal neovascularization (Figure 7). Interestingly, several miRs modulated by CRFs in PACs can directly target VEGFA (miR-15, miR-361, and miR-181) or the downstream oncogene KRAS (miR-143, miR-181), which is involved in RAS/MAPK signaling pathway. In addition, other miRs can indirectly modulate downstream factors involved in VEGF signaling or mobilization/function of PACs (Figure 7).

## Discussion

To our knowledge, this is the first comprehensive analysis of miR expression in bone marrow–derived PACs following hindlimb ischemia. It is also the first study describing the effect of CRFs on PAC miR expression profile in the context of PAD and ischemia-induced neovascularization. Although miRs are increasingly recognized as critical regulators of numerous biological and cellular processes such as growth, apoptosis, inflammation, metabolic activity, and angiogenesis (Wu, 2004; O’Connell et al., 2010; Karar and Maity, 2011; van Meeteren et al., 2011; Caporali and Emanueli, 2012; Paul et al., 2018), their specific role for the response to tissue ischemia in the context of vascular diseases is largely unknown. Moreover, how CRFs can modulate miR expression and global function in the context of angiogenesis and ischemia-induced neovascularization remains to be determined. Defining miR expression profile and modulation could be especially important in the case of bone marrow–derived PACs, whose potent angiogenic activities could be harnessed to develop novel therapies aimed at improving neovascularization and reducing tissue damage in patients with PAD. miRs could have a dual role for the modulation of PAC angiogenic properties. On the one hand, miRs have been shown to be involved in the differentiation and the functional activities of stem/progenitor cells during cardiovascular healing responses (Jakob and Landmesser, 2012). On the other hand, the angiogenic properties of PACs have been linked to their paracrine activity. In response to stress conditions such as hypoxia, PACs can release extracellular vesicles that transfer proangiogenic miRs to endothelial cells (Cantaluppi et al., 2012; Ranghino et al., 2012). Therefore, it is possible that CRFs deregulate PAC miR content and extracellular vesicle miR cargo, so that impaired angiogenic signals are transmitted in ischemic tissues. Accordingly, it becomes very important to determine miR expression profile in PACs during healing processes and investigate the modulating effects of different pathological conditions, including CRFs.

We used a well-described model of hindlimb ischemia (Limbourg et al., 2009) to investigate the effects of CRFs on the number, function, and miR expression profile of PACs. Our results demonstrate that the number and the functional activities (migration, adhesion) of PACs are impaired in CRF conditions such as aging, SMK, and HC (Figures 1B,C). We used NGS to perform a global and unbiased evaluation of PAC miR expression profile in the context of CRFs (Pritchard et al., 2012; Goodwin et al., 2016). Among 570 miRs that were found to be expressed in PACs, we focused our attention on miRs with an expression level of at least 100 RPM that showed more than 20% downregulation or 60% upregulation in a given CRF condition. We found that 40 to 61 miRs are significantly modulated by CRFs. This level of modulation is comparable to that of previous reports on the modulation pattern of miRs by CRFs in different biological fluids or tissues (Huan et al., 2018; Izzotti et al., 2018; Xu et al., 2019). For example, a recent study in human blood showed that 127 miRs (of 1,000 miRs) are differentially expressed during aging (Huan et al., 2018). Another study based on microarray analysis (a technology that does not detect all miRs) showed that 22 miRs are significantly modulated in the serum of hyperlipidemic patients (Xu et al., 2019). Also, in a mouse model, exposure to cigarette smoke was associated with a variable number of modulated miRs depending on the organ studied (Izzotti et al., 2018). Overall, these studies suggest that the number of miRs that are modulated by CRFs can vary depending on the tissue/organ studied and the detection method used. Here, although we found that the overall level of miR modulation was similar in different CRF conditions, particular effects of specific CRFs were also observed. For example, miR modulation by aging and SMK was most frequently observed in miRs with medium or high expression levels, whereas HC was also associated with the modulation of miRs that were expressed at very high levels. HC also led to more important upregulation of miRs, five miRs showing more than threefold increase compared to one and two miRs for SMK and aging, respectively. We also found that the modulation of miRs by SMK was less robust compared to that of aging and HC and that SMK was mostly associated with miR upregulation versus downregulation. Admittedly, the differences that we observed here need to be interpreted with caution, as miR modulation could depend on the intensity and/or the duration of CRF conditions. Moreover, whether the modulation of a miR that is highly expressed has more functional consequence compared to that of a miR with lower expression is currently unknown.

Our miR profiling approach in PACs identified several miRs that have previously been associated with CRFs and/or involved in related pathological conditions. For example, we found that miR-31was the most downregulated miR during aging. Interestingly, miR-31 has recently been proposed to be a valuable plasma-based biomarker for aging (Weilner et al., 2016), and it is also associated with cardiovascular diseases and hypertension (Sekar et al., 2017). Moreover, miR-31 was shown to protect against reactive oxygen species (ROS) (Stepicheva and Song, 2016). A loss of the adaptive response to oxidative stress is one of the major characteristics of aging (Finkel and Holbrook, 2000), and systemic increases of ROS levels are associated with impaired PAC function (Haddad et al., 2009; Groleau et al., 2010). In that matter, we found that miR-23b, which targets the antioxidant enzyme superoxide dismutase (Long et al., 2017), was increased by 300% in PACs during aging. Therefore, age-dependent increase of miR-23b levels could lead to defective antioxidant activity, higher ROS levels, and impaired angiogenic activities of PACs. In fact, in each CRF condition, we identified downregulated proangiogenic miRs and upregulated antiangiogenic miRs that could contribute to explain PAC dysfunction in the context of aging, SMK, and HC (Figures 3E, 4E, and 5E). Taking into account both the abundance and the degree of modulation of selected miRs in a given CRF condition, KEGG cluster connection analyses identified several pathways that could impact on PAC function (Figures 3D, 4D, and 5D). Interestingly, all CRF conditions were associated with modulation of miRs involved in cancer (Lee and Dutta, 2009) and ECM remodeling (Piccinini and Midwood, 2014), both conditions being closely linked to angiogenesis (Sottile, 2004). The central HIF1–VEGF angiogenic pathway was also shown to be targeted, together with important downstream pathways involved in VEGF signaling such as MAPK and PI3K (Ferrara et al., 2003). TGFb pathway, which regulates cellular migration, proliferation, and angiogenesis (Bertolino et al., 2005), was also shown to be involved in the three CRF conditions.

An interesting finding in the current study is that a group of miRs appears to be similarly altered in PACs exposed to different CRF conditions. For example miR-542-3p and miR-29 were decreased in all three CRF conditions, being also the two most downregulated miRs in SMK and HC conditions. Recently, miR-542-3p has been shown to contribute to the angiogenic properties of EPCs by stimulating the expression of ANGPT2 (Lu et al., 2019). On the other hand, although downregulation of miR-29c has been shown to promote ischemic brain damage via derepression of its target DNMT3a (Pandi et al., 2013), the targets of miRs can be modulated according to cell types and microenvironmental conditions. Therefore, the specific effect of miR-29c downregulation for the modulation of PAC function in PAD remains to be determined. We also identified several miRs that were upregulated in different CRF conditions. For example, miR-501-3p was the most upregulated miR in all three CRF conditions. Although the role of miR-501-3p in ischemic vascular diseases and angiogenesis has not been investigated, it is interesting to note that overexpression of miR-501-3p was shown to inhibit proliferation, migration, and invasion in cancer cells (Luo et al., 2018). Another miR that was strongly increased in all CRF conditions is miR-92a. Mir-92a has been shown to inhibit angiogenesis *in vitro* and *in vivo*, whereas inhibition of miR-92a expression can improve neovascularization and protect against ischemia in mouse ischemic hindlimbs (Bonauer et al., 2009) and after myocardial infarction in pigs (Hinkel et al., 2013). Moreover, inhibition of miR-92a can also protect PACs (EPCs) from hypoxia- or hyperglycemia-induced injury (Huang et al., 2019). We used pathway cluster dendrogram analyses to evaluate the potential resulting biological effects of miRs that are modulated in several CRF conditions. Our results indicate that the predictive pathways that are affected have key roles for the modulation of angiogenesis and PAC function, including ECM remodeling, PI3K/AKT/MAPK signaling, TGFb pathway, p53, and cell cycle progression. When looking specifically at VEGF pathway, which is crucial both for developmental and postnatal neovascularization (Ferrara et al., 2003), we found that several miRs that are modulated by CRF conditions can negatively influence VEGF-dependent angiogenesis and PAC function (Figure 7). The mechanism by which different CRFs can similarly modulate miRs involved in the angiogenic function of PACs remains to be determined. A common feature of all these CRFs is increased oxidative stress (Kunsch and Medford, 1999), which has also been associated with impaired ischemia-induced neovascularization and PAC function (Haddad et al., 2009; Groleau et al., 2010). Additional studies are needed to determine the specific role of oxidative stress for the modulation of miR expression in the context of PAC function, angiogenesis, and ischemia-induced neovascularization.

The current study has important limitations. Using NGS, we identified several miRs that were modulated in PACs in the context of CRFs, but it was not possible to confirm each miR (i.e., quantitative reverse transcription–polymerase chain reaction) in biological replicates exposed to the different conditions. In addition, targets and pathways modulated by miRs were identified using bioinformatic analyses, but we could not confirm all these results at the mRNA and protein levels in the different experimental conditions. Accordingly, the present work should be taken as a hypothesis-generating study, and the specific miRs and targets we describe in each specific experimental condition will need to be confirmed by additional experiments in future studies. We chose to study the effect of aging, SMK, and HC because those are important classical CRFs associated with impaired neovascularization and PAC dysfunction. Additional studies are needed to address the potential role of other CRFs (e.g., diabetes, hypertension), which could also modulate the expression of miRs related to PAC function. Finally, although we propose that modulating miR expression in PACs could improve their angiogenic activity and promote neovascularization in patients with PAD and CLI, it is important to note that the same miRs could have opposite and/or detrimental effects in patients who also present with other pathologies, such as cancer. Therefore, several questions remain to be answered before this type of angiogenic therapy can be developed, including which miRs to target in PACs and how to administer these “engineered” PACs in terms of dosing and delivery method (local versus systemic).

In conclusion, this study describes for the first time the effects of CRFs on the modulation of miR profile in PACs related to PAD and ischemia-induced neovascularization. We found that a significant proportion of miRs are modulated in PACs exposed to different CRFs. In each CRF condition, we identified specific proangiogenic miRs that were downregulated and also antiangiogenic miRs that were upregulated. Moreover, a group of angiogenesis-modulating miRs was found to be similarly altered in all CRF conditions. Our findings constitute a solid framework for the identification of miRs that could be targeted in PACs in order to improve their angiogenic function. In novel therapeutic strategies, these reprogrammed PACs could eventually serve as potent angiogenic vectors in order to improve neovascularization and reduce tissue damage in patients with severe PAD and CLI.

## Data Availability Statement

Next Generation Sequencing (NGS) data have been deposited and are available on NCBI Gene Expression Omnibus, GSE151609.

## Ethics Statement

The animal study was reviewed and approved by Comité Institutionnel de Protection des Animaux (CIPA) of the Centre Hospitalier de l’Université de Montréal (CHUM).

## Author Contributions

MD and AR conceptualized and designed the study. MD prepared the initial draft of the manuscript and figures. JR and AR revised the manuscript and figures. MD and SD conducted the experiments. MD, SD, and AR analyzed the data. SC provided expert advice and recommendations. All authors read and approved the final manuscript.

## Conflict of Interest

The authors declare that the research was conducted in the absence of any commercial or financial relationships that could be construed as a potential conflict of interest.

## Footnotes

1 http://mirsystem.cgm.ntu.edu.tw/index.php

2 http://www.microrna.gr/miRPathv3
