# Supplementary material for: MicroRNA Expression Profiling of Bone Marrow–Derived Proangiogenic Cells (PACs) in a Mouse Model of Hindlimb Ischemia: Modulation by Classical Cardiovascular Risk Factors
Source: Front Genet. 2020 Aug 21;11:947. doi: 10.3389/fgene.2020.00947 (PMC7472865; doi:10.3389/fgene.2020.00947)
Supplement: Supplementary file 1 [file Data_Sheet_1.PDF]

READ SET	CTL	AGING	SMK	HC
total_reads	5 569 870	6 079 301	5 198 156	5 159 332
no_adapter_reads	79 349	128 470	85 982	104 449
too_short_reads	783 181	1 311 405	887 227	864 511
umi_defective_reads	45 299	181 339	90 771	248 996
mirna_reads	3 140 294	2 325 729	2 169 192	1 794 976
hairpin_reads	2 836	4 739	4 244	5 205
pirna_reads	13 624	23 727	19 442	20 263
rrna_reads	6 023	17 025	10 285	16 258
trna_reads	915 759	1 157 427	1 155 578	1 123 386
mrna_reads	14 464	14 409	13 983	13 260
Other_rna_reads	422 408	652 143	556 838	682 754
notcharacterized_mappable	66 918	109 498	80 473	100 386
notcharacterized_notmappable	79 715	153 390	124 141	184 888

Supplemental Table 1. Summary of next generation sequencing raw reads generated
